# Prognostic significance of programmed death-1 and programmed death-ligand 1 expression in patients with esophageal squamous cell carcinoma

**DOI:** 10.18632/oncotarget.8956

**Published:** 2016-04-23

**Authors:** Kaiyan Chen, Guoping Cheng, Fanrong Zhang, Nan Zhang, Dan Li, Jiaoyue Jin, Junzhou Wu, Lisha Ying, Weimin Mao, Dan Su

**Affiliations:** ^1^ Cancer Research Institute, Zhejiang Cancer Hospital and Key Laboratory Diagnosis and Treatment Technology on Thoracic Oncology of Zhejiang Province, Hangzhou, China; ^2^ Department of Oncology, The Second Clinical Medical College of Zhejiang Chinese Medical University, Hangzhou, China; ^3^ Department of Pathology, Zhejiang Cancer Hospital, Hangzhou, China

**Keywords:** esophageal cancer, PD-1, PD-L1, immunochemistry, prognosis

## Abstract

**Aims:**

To evaluate the expression of programmed death-1 (PD-1) and programmed death-ligand 1 (PD-L1) and their clinical and prognostic significance in primary esophageal squamous cell carcinoma (ESCC).

**Results:**

The expression rate of PD-1 and PD-L1 in ESCC was 33.5% (117/349) and 41.4% (222/536), respectively. PD-L1 expression differed significantly by tumor location, grade, lymph node metastases, and disease stage (*P* < 0.05). Moreover, its expression was associated with the disease free survival (DFS). Patients with positive PD-L1 expression had reduced risk for disease relapse compared to those without PD-L1 expression (Hazard ratio [HR] = 0.75, 95% confidence interval [CI]: 0.56–1.00, *P* = 0.048). Kaplan-Meier curves showed the similar result, *P* = 0.047. However, there was no significant correlation between PD-1 expression and clinicopathological factors or outcome in ESCC (*P* > 0.05).

**Methods:**

The expression of PD-1 and PD-L1 was assessed by immunohistochemistry on tissue microarrays from 536 primary ESCC who underwent surgery during January 2008 and April 2012 in Zhejiang Cancer Hospital. Chi-square test and Cox proportional hazards regression were employed to analyze the associations between their expressions and clinicopathological variables and survival.

**Conclusions:**

Our results suggested that PD-L1 could be a favorable indicator of prognosis in ESCC.

## INTRODUCTION

Esophageal carcinoma is one of the most aggressive carcinomas, which is the sixth leading cause of cancer-related mortality worldwide [1]. Approximate, 70% of global esophageal cancer cases occur in China [2], and 90% of them are esophageal squamous cell carcinoma (ESCC) [3]. Despite recent improvements in therapy, the outcome of ESCC still remains poor with 5-year overall survival rate of 25% to 40% resulted from local recurrence or distant metastasis [4].

Immune resistance plays an important role in the initiation and development of many malignant tumors, including ESCC [5, 6]. Costimulatory signaling has been implicated as one of the potential immune resistance mechanisms, which is critical for the regulation of T-cell activation [7]. Programmed death-1 (PD-1), one of the negative costimulatory molecules, is the key immune checkpoint receptor to inhibit T-cell activation [5]. Belonging to the CD28 family, it is expressed on tumor-infiltrating lymphocytes (TILs) including T cells, B cells, and myeloid cells [7]. Two ligands for PD-1, PD-L1 (also known as B7-H1) and PD-L2 (also known as B7-DC) have been identified. They belong to the B7 family of immune-regulatory ligands and both can be found on tumor cells or stromal cells, as well as typical antigen presenting cells [8, 9]. Previous studies showed that the expression of PD-1 and PD-L1 was correlated with impaired immune responses and worsen prognosis in various cancers [10–13]. And patients with malignant melanoma, non-small cell lung cancer (NSCLC), or renal cell carcinoma are beneficial from anti-PD-1 and anti-PD-L1 therapy [5, 10, 14, 15]. There is indication that PD-1 or PD-L1 may be a predictive biomarker for treatment response [16]. However, recent literatures reported that patients with high expression levels in PD-1 and PD-L1 had better prognosis in breast cancer, glioblastoma, metastatic melanoma, colorectal cancer, pulmonary squamous cell carcinoma, and ovarian cancer [15, 17–20]. The role of PD-1 and PD-L1 expression in various solid tumors remains controversial.

Few studies mentioned the prognostic relevance of PD-1 and PD-L1 expression in ESCC. Recently, two studies demonstrated that the PD-L1 expression was relevant to worse prognosis in ESCC [4, 21]. Nonetheless, the two cohorts were relatively small and did not provide reliable observations. In the present study, we systematically investigated the expression of PD-1 and PD-L1 on 536 ESCC tissue samples and analyzed their association with the clinicopathological characteristics and prognosis.

## RESULTS

### Expression of PD-L1 and PD-1

**Figure 1:**
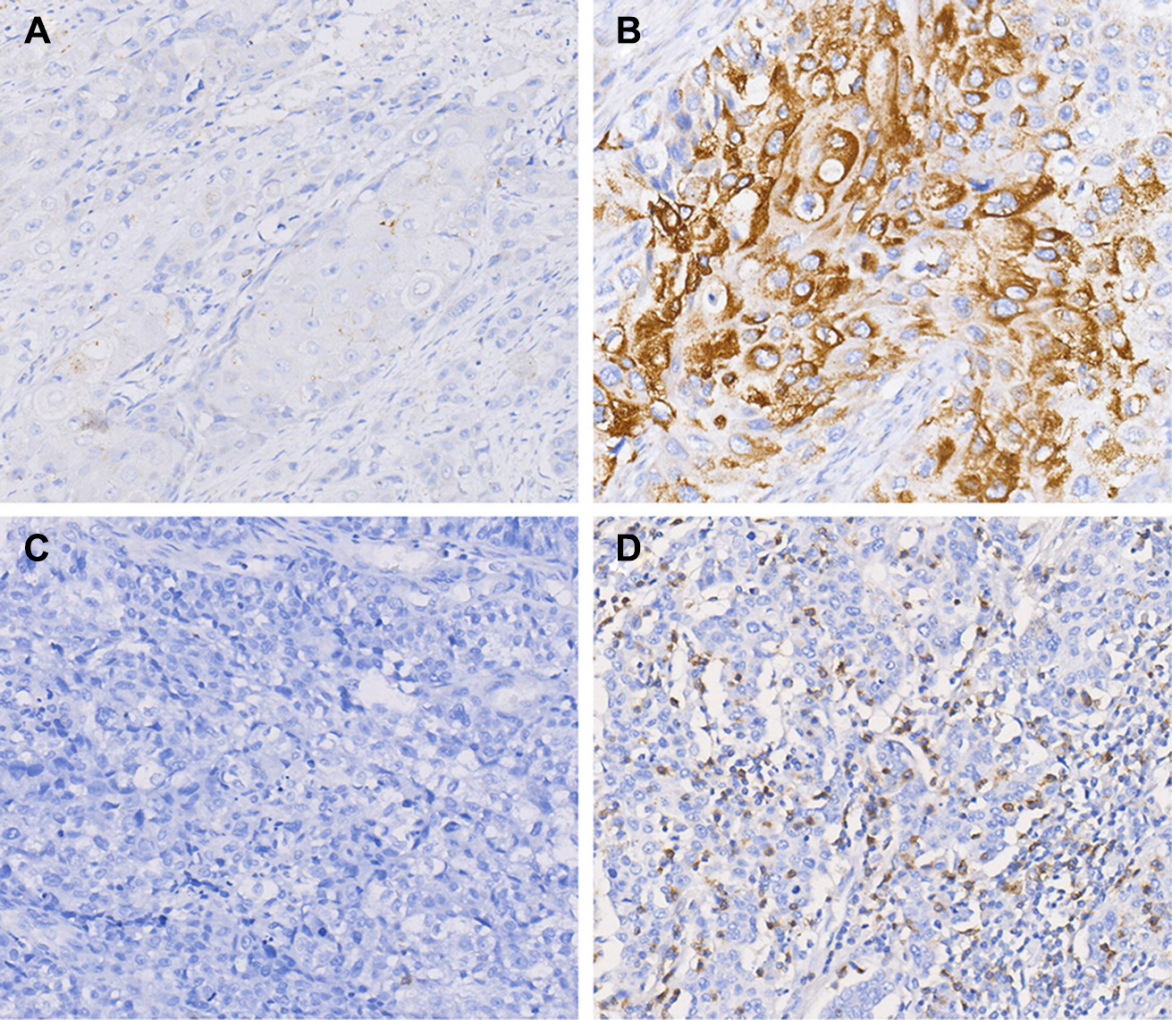
PD-1 and PD-L1 expression in ESCC by immunohistochemistry staining (**A**) Negative expression of PD-L1. (**B**) Positive expression of PD-L1. (**C**) Negative expression of PD-1. (**D**) Positive expression of PD-1. Original magnification ×200.

PD-L1 was found to be located on both the membrane and in the cytoplasm of cancer cells. Figure 1 showed the representative images for PD-L1 staining negative (A), positive (B). In total, 41.4% (222/536) of ESCC patients showed the positive expression of PD-L1 in tumor cells. PD-1 was found expressed on the cell membrane of TILs in 33.5% (117/349) of ESCC. The representative images for PD-1 staining were shown in Figure 1.

### Clinicopathological associations of PD-L1 and PD-1 expression

The clinicopathological characteristics of the patients and their association with PD-L1 protein expression are summarized in Table 1. Patients' median age at diagnosis was 60 years, with a range of 37 to 77 years. Among the 536 patients, 394 (73.5%) were younger than 65 years, 464 (86.0%) were male, 138 (25.7%) had family history, 382 (71.3%) had alcohol drinking and 403 (75.2%) had smoking experience. Within the cohort, 35 cases (6.5%) had well differentiated tumor, 377 (70.3%) moderately differentiated and 124 (23.1%) poorly differentiated. According to the 7th IUCC/AJCC staging system, 61 (11.4%) patients were stage I, 195 (36.4%) patients were stage II, 273 (50.9%) patients were stage III, and 7 (1.3%) patients were stage IV. Statistical analysis indicated that the upper esophageal location, better tumor differentiation, negative nodal (N) stage, and early tumor stage were correlated with the positive expression of PD-L1 (*P <* 0.05). There was no significant correlation between PD-L1 expression and the age, gender, tumor (T) stage, family history, alcohol drinking, smoking experience, and Body Mass Index (BMI) (*P >* 0.05).

**Table 1:** Association of PD-L1 expression with clinicopathological factors in 536 ESCC patients

Category	All cases	PD-L1		*P*-value
		+	−	
**Age**				
< 65	394 (73.5%)	163 (41.4%)	231 (58.6%)	0.970
≥ 65	142 (26.5%)	59 (41.5%)	83 (58.5%)	
**Gender**				
Male	464 (86.0%)	193 (41.6%)	271 (58.4%)	0.833
Female	72 (14.0%)	29 (40.3%)	43 (59.7%)	
**Tumor site**				
Upper	17 (3.2%)	7 (41.2%)	10 (58.8%)	***0.037***
Middle	155 (28.9%)	51 (32.9%)	104 (67.1%)	
Lower	364 (67.9%)	164 (45.1%)	200 (54.9%)	
**Differentiation**				
Well	35 (6.5%)	19 (54.3%)	16 (45.7%)	***0.010***
Moderate	377 (70.3%)	165 (43.8%)	212 (56.2%)	
Poor	124 (23.1%)	38 (30.6%)	86 (69.4%)	
**T**				
T1	28 (5.2%)	14 (50.0%)	14 (50.0%)	0.204
T2	92 (17.2%)	43 (46.7%)	49 (53.3%)	
T3	405 (75.6%)	163 (40.2%)	242 (59.8%)	
T4	11 (2.1%)	2 (18.2%)	9 (81.8%)	
**N**				
N0	222 (41.4%)	115 (51.8%)	107 (48.2%)	***0.000***
N1	170 (31.7%)	55 (32.4%)	115 (67.6%)	
N2	107 (20.0%)	42 (39.3%)	65 (60.7%)	
N3	37 (6.9%)	10 (27.0%)	27 (73.0%)	
**TNM stage**				
I	61 (11.4%)	35 (57.4%)	26 (42.6%)	***0.002***
II	195 (36.4%)	90 (46.2%)	105 (53.8%)	
III	273 (50.9%)	93 (34.1%)	180 (65.9%)	
IV	7 (1.3%)	4 (57.1%)	3 (42.9%)	
**Family history**				
Yes	138 (25.7%)	55 (39.9%)	83 (60.1%)	0.665
No	398 (74.3%)	167 (42.0%)	231 (58.0%)	
**Alcohol**				
Yes	382 (71.3%)	157 (41.1%)	225 (58.9%)	0.814
No	154 (28.7%)	65 (42.2%)	89 (57.8%)	
**Smoking**				
Yes	403 (75.2%)	166 (41.2%)	237 (58.8%)	0.853
No	133 (24.8%)	56 (42.1%)	77 (57.9%)	
**BMI**				
< 18	71 (13.2%)	36 (50.7%)	35 (49.3%)	0.189
18–25	412 (76.9%)	167 (40.5%)	245 (59.5%)	
> 25	53 (9.9%)	19 (35.8%)	34 (64.2%)	
**Total**	**536**	**222 (41.4%)**	**314 (58.6%)**	

a Bold-italic values are statistically significant (*p <* 0.05).

PD-1 expression had no significant correlation with any clinicopathological factors examined (*P >* 0.05), as shown in Table 2.

**Table 2:** Association of PD-1 expression with clinicopathological factors in 349 ESCC patients

Category	All cases	PD-1		*P*-value
		+	−	
**Age**				
< 65	91 (26.1%)	34 (37.4%)	57 (62.6%)	0.367
≥ 65	258 (73.9%)	83 (32.2%)	175 (67.8%)	
**Gender**				
Male	295 (84.5%)	97 (32.9%)	198 (67.1%)	0.552
Female	54 (15.5%)	20 (37.0%)	34 (63.0%)	
**Tumor location**				
Upper	12 (3.4%)	2 (16.7%)	10 (83.3%)	0.339
Middle	98 (28.1%)	31 (31.6%)	67 (68.4%)	
Lower	239 (68.5%)	84 (35.1%)	155 (64.9%)	
**Differentiation**				
Well	22 (6.3%)	5 (22.7%)	17 (77.3%)	0.054
Moderate	237 (67.9%)	73 (30.8%)	164 (69.2)	
Poor	90 (25.8%)	39 (43.3%)	51 (56.7%)	
**T**				
T1	22 (6.3%)	5 (22.7%)	17 (77.3%)	0.226
T2	62 (17.8%)	26 (41.9%)	36 (58.1%)	
T3	257 (73.6%)	82 (31.9%)	175 (68.1%)	
T4	8 (2.3%)	4 (50.0%)	4 (50.0%)	
**N**				
N0	147 (42.1%)	48 (32.7%)	99 (67.3%)	0.694
N1	104 (29.8%)	33 (31.7%)	71 (68.3%)	
N2	73 (20.9%)	25 (34.2%)	48 (65.8%)	
N3	25 (7.2%)	11 (44.0%)	14 (56.0%)	
**TNM stage**				
I	41 (11.7%)	14 (34.1%)	27 (65.9%)	1.000
II	131 (37.5%)	44 (33.6%)	87 (66.4%)	
III	174 (49.9%)	58 (33.3%)	116 (66.7%)	
IV	3 (0.9%)	1 (33.3%)	2 (66.7%)	
**Family history**				
Yes	97 (27.8%)	28 (28.9%)	69 (71.1%)	0.253
No	252 (72.2%)	89 (35.3%)	163 (64.7%)	
**Alcohol**				
Yes	242 (69.3%)	79 (32.6%)	163 (67.4%)	0.601
No	107 (30.7%)	38 (35.5%)	69 (64.5%)	
**Smoking**				
Yes	255 (73.1%)	81 (31.8%)	174 (68.2%)	0.251
No	94 (26.9%)	36 (38.3%)	58 (61.7%)	
**BMI**				
< 18	48 (13.8%)	16 (33.3%)	32 (66.7%)	0.770
18–25	265 (75.9%)	87 (32.8%)	178 (67.2%)	
> 25	36 (10.3%)	14 (38.9%)	22 (61.1%)	
**Total**	**349**	**117 (33.5%)**	**232 (66.5%)**	

### Prognostic effect of PD-L1 and PD-1 expression

**Figure 2:**
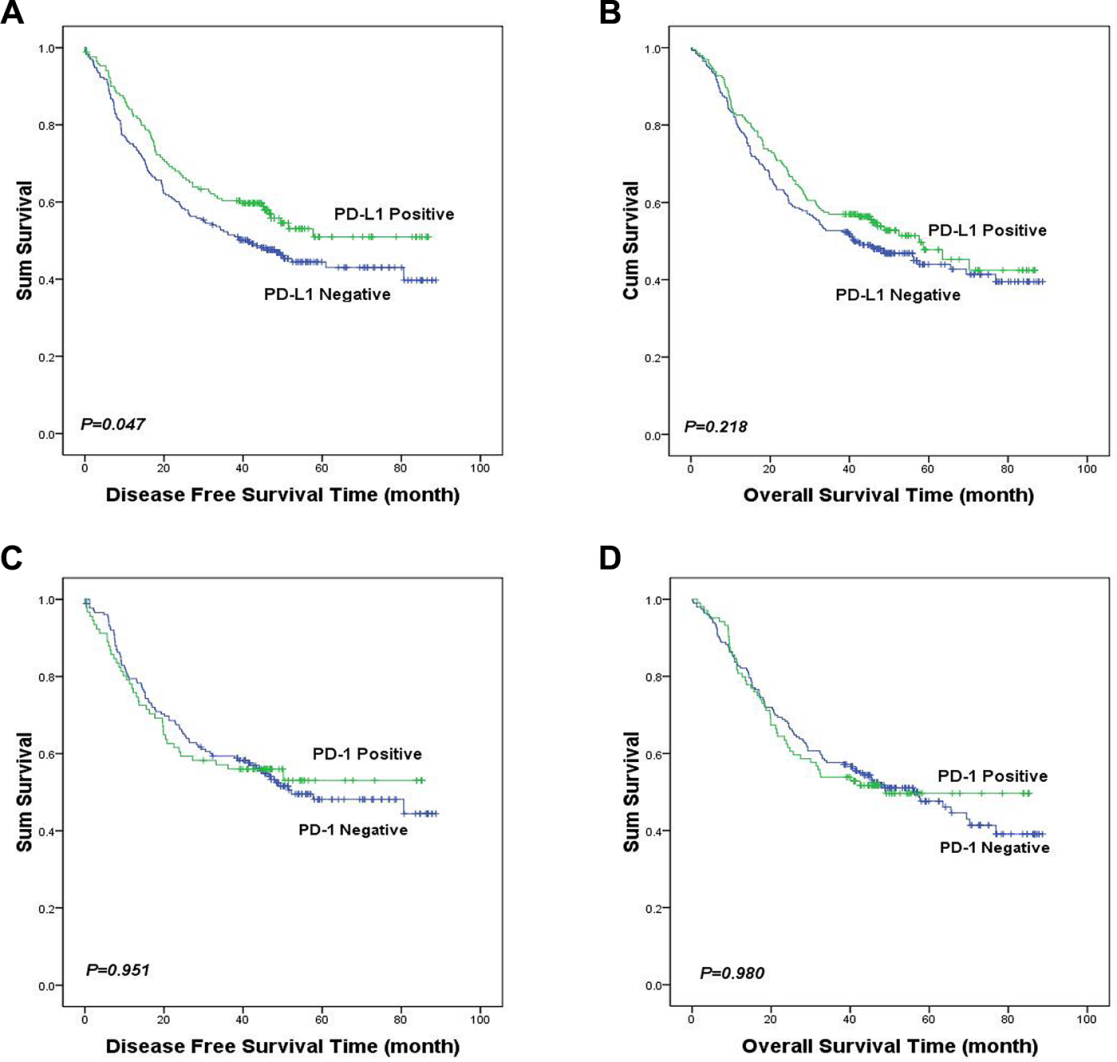
Kaplan-Meier curves of DFS and OS in ESCC based upon PD-1 and PD-L1 expression Patients with PD-L1 expression had significantly longer DFS than those without PD-L1 expression (median DFS: not reached verse 41.3 months, *P* = 0.047, (**A**). There was no statistically significant difference in OS between the patients with positive and negative PD-L1 staining (median OS: 57.6 verse 41.3 months, *P* = 0.218, (**B**). PD-1 expression was not significantly correlated with DFS or OS in ESCC (*P >* 0.05, (**C** and **D**).

As shown in Table 3, univariate analysis demonstrated that patients with PD-L1 expression had lower risk to relapse than those with no expression (Hazard ratio [HR] = 0.75, 95% confidence interval [CI]: 0.56–1.00, *P* = 0.048). However, multivariate analysis failed to suggest PD-L1 be an independent prognostic factor (HR = 0.80, 95% CI: 0.55–1.17, *P* = 0.249 for disease free survival [DFS]; HR = 0.83, 95% CI: 0.59–1.18, *P* = 0.293 for overall survival [OS]). Kaplan-Meier analysis showed the similar results (Figure 2). The median DFS of the patients with PD-L1 expression was significantly longer than the patients without PD-L1 expression (not reached verse 41.3 months, *P* = 0.047). Same tendency was found for OS that the median OS of patients with PD-L1 expression was 57.6 months compared with 41.3 months for patients without. However, the difference was not statistically significant (*P* = 0.218).

**Table 3:** Univariate and multivariate cox regression analyses estimating the associations of PD-1 and PD-L1 expression with patient survival

	Crude HR	95%CI	*P*-value	Adjust HR	95%CI	*P*-value
**Disease free survival**						
**PD-1**						
Negative	1.00			1.00		
Positive	0.99	0.68–1.43	0.951	0.94	0.64–1.37	0.737
**PD-L1**						
Negative	1.00			1.00		
Positive	0.75	0.56–1.00	***0.048***	0.80	0.55–1.17	0.249
**Overall survival**						
**PD-1**						
Negative	1.00			1.00		
Positive	1.05	0.80–1.37	0.748	0.87	0.62–1.24	0.452
**PD-L1**						
Negative	1.00			1.00		
Positive	0.85	0.65–1.10	0.219	0.83	0.59–1.18	0.293

a Associations determined by Cox proportional hazards regression and adjusted for age, sex, tumor site, stage, grade, smoking experience, alcohol drinking and family history.

b Bold-italic values are statistically significant (*p <* 0.05).

No significant correlation between PD-1 expression and DFS or OS was found (HR = 0.99, 95% CI: 0.68–1.43, *P* = 0.951 for DFS; HR = 1.05, 95% CI 0.80–1.37, *P* = 0.748 for OS), as shown in Table 3.

## DISCUSSION

In this study, the expression rate of PD-L1 in ESCC patients was 41.4%, which is similar to Ohigashi's study on ESCC patients (43.9%) [4]. Meanwhile, a meta-analysis demonstrated that nearly 50% of gastrointestinal tract cancer were positive for PD-L1 expression regardless the method of evaluation [22].

Interestingly, in the present study, patients with tumor that was at upper esophageal location, well differentiated, absence of lymph node metastasis, or at the early stage were more likely to have positive expression of PD-L1, suggesting that PD-L1 expression is an indicator of less aggressive tumors. However, Ohigashi's study did not find any significant correlation between PD-L1 expression and clinicopathological factors in 41 ESCC patients [4]. In another study by Chen et al. [21], PD-L1 expression was only correlated with tumor invasion in 99 patients. Moreover, in the study PD-L1 expression was detected in both membrane/cytoplasm and nucleus, while ours and others found PD-L1 expression only in the membrane/cytoplasm of cancer cells. A study of colorectal cancer showed similar results as to ours [23], in which they found a positive correlation between PD-L1 expression and early stage disease, well differentiation tumor, as well as the absence of lymph node metastasis and vascular invasion. Further studies to investigate the molecular mechanisms of these correlations are needed.

Unlike earlier studies [4, 6, 21, 24], we observed that patients with PD-L1 expression had longer relapse time and overall survival time in the first 5 years after operation compared to those without PD-L1 expression, and others found similar associations as to ours in different tumor types [20, 23, 25–27]. A study of 636 breast cancer patients found that higher PD-L1 mRNA expression was significantly associated with increased TILs and longer DFS [27]. Similarly, in colorectal cancer, strong PD-L1 expression was correlated with the infiltration of CD8-positive lymphocytes and improved OS [23]. PD-L1 was also defined to indicate favorable prognosis in pulmonary squamous cell carcinoma [28].

It is known that PD-L1 and PD-1 interaction leads to immune suppression which may partially be responsible for the immune resistance of tumor cells, but high PD-L1 expression may also promote immune responses through PD-L1's binding to unknown receptors other than PD-1, resulting in T-cell proliferation and secretion of certain cytokines such as IL-10 and interferon γ [4, 29], which in turn activate strong antitumor effects. In addition, it has been shown that localized PD-L1 expression promotes organ-specific autoimmunity [30]. Moreover, studies have shown that, in the highly dynamic tumor-immune system, the expression of PD-L1 in tumor microenvironment can also be induced by CD8-positive T cells, as well as cytokines such as interferon γ, IL-2, IL-7, IL-15, and IL-21 in a positive feedback mechanism [27, 31, 32]. The presence of CD8-positive T cell infiltration in esophageal carcinomas has been reported to be a favorable prognostic factor with potential clinical implications [33, 34].

In our study, 33.5% of ESCCs showed PD-1 expression in TIL, which was consistent with the results reported by D'Incecco1 et al. (35.2%) for NSCLC [35]. However, this observation was not in line with the previous results from studies of gastric, pancreatic and renal cell cancers [10, 11, 13]. We didn't find any association between PD-1 expression and clinicopathological factors or outcomes in ESCC. There were no previous studies that either support or contradict to our study.

To our knowledge, this is the first study that systematically evaluated the expression of PD-1 and PD-L1 and their associations with clinicopathological factors and outcome in a rather large cohort of resectable ESCC. Our findings suggested that PD-L1 expression is a favorable indicator for ESCC prognosis.

## MATERIALS AND METHODS

### Study population

Samples and clinical data for 536 primary ESCC patients who underwent surgical resection during January 2008 and April 2012 in Zhejiang Cancer Hospital, China were retrospectively studied. No patients received pre-operative chemotherapy or radiotherapy. The extent of the disease was determined by TNM staging based on the 7th IUCC/AJCC recommendations. All tissue specimens used in our study were obtained from the tissue bank of Zhejiang Cancer Hospital and all patients were provided informed consent before surgery. This study was approved by the institutional review board of Zhejiang Cancer Hospital.

Regular follow-up was performed for all patients at three-month interval after operation in the first two years, six-month interval in the third year and yearly thereafter. Follow-up evaluation includes physical examination, complete blood count, and enhanced computational tomography for chest, gastroscope and abdominal ultrasound. The median follow-up time is 32.7 months with a range from 1.0 to 88.7 months. OS were available for 451 (84.1%) patients, and among whom 261 (57.9%) patients died during the follow-ups. DFS were available for 403 (75.2%) patients, and 224 (41.8%) patients underwent disease progress, of which 190 (84.8%) patients died.

### Tissue microarray

Formalin-fixed paraffin-embedded (FFPE) tumor tissue samples were hematoxylin and eosin (H&E) stained, and ESCC was confirmed by two senior pathologists independently. The paraffin tissue blocks of 536 cases of esophageal cancer were used in the construction of tissue microarray. In brief, the H&E-stained standard slides were reviewed from each section of esophageal cancer tissues, and one representative tumor area of each tumor (2 mm diameter) were removed from FFPE tissue blocks. A serial of 3-μm-thick sections were cut for the purpose of immunohistochemistry and transferred to adhesive slides according to manufacturer's instructions.

### Immunohistochemistry

Standard immunohistochemical analysis was performed with the primary antibody against human PD-1 (clone NAT105, mouse immunoglobulin G1, Abcam, Cambridge, UK) and PD-L1 (clone SAB2900365, rabbit immunoglobulin G1, Sigma-Aldrich, Saint Louis, USA) at a dilution in 1:100 and 1:400, respectively. Briefly, antigen retrieval was achieved by microwave pretreatment in citrate buffer. After neutralization of endogenous peroxidase, tissue microarray slides were preincubated with blocking serum and then were incubated with PD-1 or PD-L1 antibody for 40 minutes at room temperature. After three washes in PBS, the slides were treated with the horseradish peroxidase (HRP)-labeled goat anti-mouse/rabbit secondary antibody (Dako, Glostrup, Denmark) for 20 minutes at room temperature, then continued to wash in PBS. Finally, reaction products were visualized with 3,3′-diaminobenzidine (DAB, Dako, Glostrup, Denmark) and the slides were counterstained with hematoxylin. After being dehydrated, slides were mounted in resin.

### Evaluation of immunohistochemical staining

Immunohistochemistry results were evaluated by scanning each slide under low power magnification (× 100) to identify regions containing positive immunoreactivity. Immunostainings were further evaluated at high power magnification (× 400). In accordance with previously published approaches [6, 28], PD-1 expression levels in lymphocytes and PD-L1 expression levels in tumor cells were as intensity assessed with immunostaining considering 0 as negative, 1 as weak, 2 as moderate and 3 as high. The tissues having no TILs were excluded from our study; hence the data of only 349 patients were used to assess the PD-1 expression level. TILs were shown in H&E–stained TMA slides and the cell counts were enumerated independently by two pathologists under the entire visual region. Then PD-1 positive area was evaluated in each histospot. The tumors were evaluated as PD-L1 positive if 5% of the tumor cells displayed at least moderate staining. The tumors were evaluated as PD-1 positive if 5% of the lymphocytes displayed at least moderate staining. Staining intensity and area of stained cells were determined independently by two senior pathologists in a blind manner, doubtful cases were discussed by the two pathologists until consensus was achieved.

### Statistical analysis

Statistical analyses were performed using statistics software (version 18.0; SPSS, Chicago, IL). Associations with clinicopathological variables were analyzed using the Pearson's chi-square or Fisher's Exact test. Associations with OS and DFS were analyzed using the Kaplan-Meier method, log-rank test and the Cox proportional hazards model. *P*-value < 0.05 in a two-tailed test was considered statistical significance.
